# Supplementary material for: Investigation of Brucella melitensis in Sable Antelope (Hippotragus niger) in South Africa
Source: Microorganisms. 2020 Sep 29;8(10):1494. doi: 10.3390/microorganisms8101494 (PMC7600299; doi:10.3390/microorganisms8101494)
Supplement: Supplementary file 1 [file microorganisms-08-01494-s001.pdf]

Supplementary Data 1: Mitigation plan for Farm 1

Positive sable in isolation

1. Put down the positive cows as they have no future and will always be positive and infective to other livestock and need to be destroyed. Take photographic evidence with witnesses and collect material for genetic and possible traceback purposes as soon as possible.
2. Positive bulls are to be kept separate from other livestock and sable and allowed to be shot as trophies on the farm. Material also to be collected for genetic determination.

Rest of sable

Group A:

a. All positive cases are to be removed and in isolation.
b. Test the whole group and offspring in as short a time as possible.
c. All animals are to be ear-tagged and microchipped if not already done. If all tested negative, then it will be regarded as being a negative herd after testing a second time.
d. Considering some positives came from this group, the negative animals would still be kept as a group and retested after they have calved.
e. Any positives, if they occur, would be destroyed if female. Male positive cases would be kept for trophy hunting in four to five years' time in a separate group.

Group B:

f. As this group only had positive cows and offspring, which were removed in April 2004, it is assumed that they are clean at this stage.
g. The plan is to test the whole group as soon as possible plus offspring.
h. If all tested negative, they would be retested in six months' time.
i. Should they all test negative in six months, group B could be regarded as brucellosis-free and kept as such.

Group C:

j. This group of animals were already tested and put into a boma about two months before brucellosis infection was confirmed on the farm.
k. One cow is heavily pregnant and will need to be tested as soon as she calves as she originally came from group A and is ex-Farm 2.
l. The whole group would be tested. If all negative, it would be made available for sale, as sable which have been tested twice (while in isolation in the boma) for brucellosis with negative results can be certified as brucellosis-free.

Animals Sold

1. Follow up on what has happened to animals sold and to test them as soon as possible.
2. Contact the state veterinarian in charge of Farm 3 and make the veterinarian aware that one of the animals bought from them had tested positive for brucellosis and to let the breeder know of the possibility this animal may have been positive when sold, as a warning.

State Quarantine Restrictions on the Sable Antelopes as part of the mitigation plan

1. In future, all sable that are immobilised to move or be treated will be bled and tested for brucellosis to make sure herds remain clean. As the owner plans to move the sable to the large game camps, this could be done at the same time.

2. Any animal to be sold or removed off the property will be tested first to see it is still negative. This will also protect the farmer against any possible comeback in the future.
3. The two young positive brucellosis sable bulls must always stay in isolation, never leave the farm, and authorities must be notified when they have been destroyed.
4. The costs of testing will be covered by the Department of Veterinary Services and will include laboratory costs and visitation by officials. (This does not include immobilisation, drugs used, etc.)
5. Any introductions of sable will first be tested for brucellosis. If free, they will be quarantined on the farm and retested before they are allowed free. This is to protect this farm from reintroducing the disease.
6. Once the two positive brucellosis bulls have been destroyed, the situation will be reviewed and failing any detection of disease, the quarantine will be completely lifted on the farm. The plan is that the quarantine will remain in force until the remaining animals are tested in six months' time. If they continue to test negative, the quarantine should be able to be lifted.

Veterinary Officers Intervention

1. The state veterinarian undertook the contacting of a colleague to obtain his perspective and to see if he was prepared to write an article in SA Wild and Wildlife magazine in order to advise all potential purchasers of roan and sable to have them tested for brucellosis before purchase. This would soon help to see the extent of the disease as well as to protect the buyer, as it is suspected that the disease is far more widespread in game than anyone realises.
2. The state veterinarian was to contact his colleague to find out more of what is happening within Farm 2, to inquire if *Brucella* was cultured from seropositive buffaloes at Farm 2, and to inquire on the course of action.
3. The state veterinarian will write out a test protocol of what they were planning to do to get the sable free of the infection so he can sell sable again.

Measures to reduce herd transmission

1. Brucellosis-positive cows need to be kept in complete isolation with no other cloven-hooved animals coming in contact with them.
2. The bulls can be shot as trophies but will always have to be isolated from the other sable.
3. The farm must be tested until two consecutive negative tests for brucellosis of all animals
